# *N*-[2-(4-Bromo­benzo­yl)eth­yl]isopropyl­aminium chloride

**DOI:** 10.1107/S1600536811052640

**Published:** 2011-12-10

**Authors:** Abdullah Aydın, Mehmet Akkurt, Halise Inci Gul, Ebru Mete, Ertan Sahin

**Affiliations:** a Department of Science Education, Faculty of Education, Kastamonu University, 37200 Kastamonu, Turkey; b Department of Physics, Faculty of Sciences, Erciyes University, 38039 Kayseri, Turkey; c Department of Pharmaceutical Chemistry, Faculty of Pharmacy, Atatürk University, 25240 Erzurum, Turkey; d Department of Chemistry, Faculty of Sciences, Atatürk University, 25240 Erzurum, Turkey

## Abstract

The crystal structure of the title compound, C_12_H_17_BrNO^+^·Cl^−^, is stabilized by N—H⋯Cl and C—H⋯O hydrogen bonds, forming a three-dimensional network. The inter­actions framework is completed by C—H⋯π contacts between a methyl­ene group and the benzene ring of a symmetry-related mol­ecule.

## Related literature

For details of the pharmacological effects of Mannich bases and for the synthesis, see: Dimmock & Kumar (1997 ▶); Gul, Gul, *et al.* (2005 ▶); Gul, Sahin *et al.* (2005 ▶); Gul *et al.* (2007 ▶); Mete *et al.* (2011 ▶); Kucukoglu *et al.* (2011 ▶); Canturk *et al.* (2008 ▶); Chen *et al.* (1991 ▶); Suleyman *et al.* (2007 ▶). For bond-length data, see: Allen *et al.* (1987 ▶).
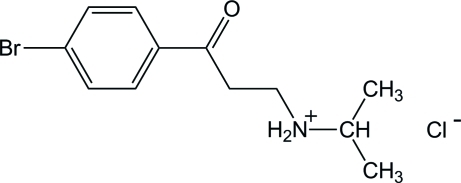


## Experimental

### 

#### Crystal data

C_12_H_17_BrNO^+^·Cl^−^
*M* *_r_* = 306.62
Tetragonal, 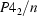

*a* = 19.7122 (4) Å
*c* = 7.1738 (2) Å
*V* = 2787.53 (11) Å^3^
*Z* = 8
Mo *K*α radiation
μ = 3.12 mm^−1^
*T* = 294 K
0.15 × 0.13 × 0.11 mm

#### Data collection

Rigaku R-AXIS RAPID-S diffractometer
Absorption correction: multi-scan (Blessing, 1995 ▶) *T*_min_ = 0.632, *T*_max_ = 0.709
50060 measured reflections
2836 independent reflections
1617 reflections with *I* > 2σ(*I*)
*R*_int_ = 0.151

#### Refinement

*R*[*F* ^2^ > 2σ(*F* ^2^)] = 0.076
*wR*(*F* ^2^) = 0.181
*S* = 1.07
2836 reflections
153 parameters
2 restraints
H atoms treated by a mixture of independent and constrained refinement
Δρ_max_ = 0.67 e Å^−3^
Δρ_min_ = −0.81 e Å^−3^

### 

Data collection: *CrystalClear* (Rigaku/MSC, 2005 ▶); cell refinement: *CrystalClear*; data reduction: *CrystalClear*; program(s) used to solve structure: *SIR97* (Altomare *et al.*, 1999 ▶); program(s) used to refine structure: *SHELXL97* (Sheldrick, 2008 ▶); molecular graphics: *ORTEP-3 for Windows* (Farrugia, 1997 ▶) and *PLATON* (Spek, 2009 ▶); software used to prepare material for publication: *WinGX* (Farrugia, 1999 ▶).

## Supplementary Material

Crystal structure: contains datablock(s) global, I. DOI: 10.1107/S1600536811052640/bh2400sup1.cif

Structure factors: contains datablock(s) I. DOI: 10.1107/S1600536811052640/bh2400Isup2.hkl

Supplementary material file. DOI: 10.1107/S1600536811052640/bh2400Isup3.cml

Additional supplementary materials:  crystallographic information; 3D view; checkCIF report

## Figures and Tables

**Table 1:** Hydrogen-bond geometry (Å, °) *Cg*1 is the centroid of the benzene ring.

*D*—H⋯*A*	*D*—H	H⋯*A*	*D*⋯*A*	*D*—H⋯*A*
N1—H*N*2⋯Cl1	0.86 (4)	2.26 (4)	3.102 (4)	166 (5)
N1—H*N*1⋯Cl1^i^	0.86 (6)	2.27 (6)	3.133 (5)	177 (9)
C12—H12*B*⋯O1^ii^	0.96	2.60	3.378 (7)	139
C9—H9*B*⋯*Cg*1^iii^	0.97	3.00	3.943 (6)	164

Symmetry codes: (i) 
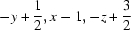
; (ii) 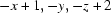; (iii) 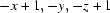.

**Fig. 1.**
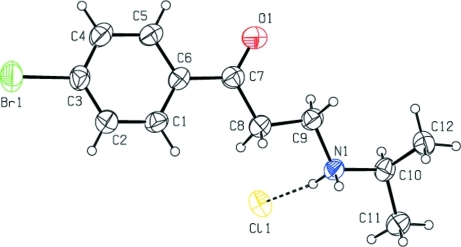
The title compound with displacement ellipsoids for non-H atoms shown at the 30% probability level.

**Fig. 2.**
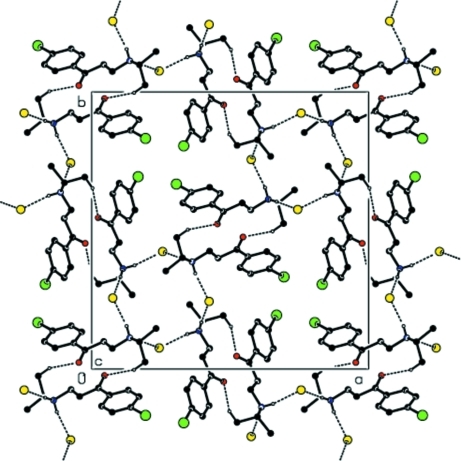
The packing and hydrogen bonding of the title salt viewed down the c axis. H atoms not involved in hydrogen bonds are omitted for the sake of clarity.

## supplementary crystallographic information

### Comment

Mannich bases are generally formed by the reaction between formaldehyde, a secondary amine and a compound containing reactive hydrogen atoms. On occasion, aldehydes other than formaldehyde may be employed and the secondary amine may be replaced by ammonia and primary amines. This process is known as the Mannich reaction (Dimmock & Kumar, 1997).

Mannich bases display varied biological activities such as antimicrobial (Gul, Sahin, *et al.*, 2005), cytotoxic (Gul, Gul *et al.*, 2005; Gul *et al.*, 2007; Mete *et al.*, 2011; Kucukoglu *et al.*, 2011), anticancer (Dimmock & Kumar, 1997; Chen *et al.*, 1991), anti-inflammatory (Suleyman *et al.*, 2007), and DNA topoisomerase I inhibiting properties (Canturk *et al.*, 2008).

A Mannich base having at least one hydrogen atom at the *β* position of amine group can undergo a deamination process to generate an *α*,*β*-unsaturated ketone moiety.

In the molecule of the tile compound (Fig. 1), the bond lengths are within normal ranges (Allen *et al.*, 1987), as well as bond angles.

In the crystal structure, molecules are linked *via* intermolecular N—H···Cl and C—H···O hydrogen bonds (Table 1, Fig. 2), forming a three dimensional network. Furthermore, a C—H···π interaction (Table 1) contributes to the stabilization of the crystal packing.

### Experimental

A mixture of the appropriate ketone (50 mmol), *para*-formaldehyde (50 mmol), and *iso*-propylamine hydrochloride (27 mmol) was heated in an oil bath at 403 K. The reaction vessel was then removed from the oil bath and when the temperature of the mixture dropped to 338 K, ethyl acetate (40–80 ml) was added. The mixture was stirred at room temperature for 24 h. and the resulting precipitates were then collected and the Mannich base (I) was passed through a column of silica gel 60 (70–230 mesh) using methanol as eluent. After evaporation of the solvent, the product was recrystallized from methanol. *M*.p.: 447–449 K. Yield: 38%. ^1^H-NMR (CDCl_3_) δ 1.49 (d, *J* = 6.8 Hz, 6H, CH(CH_3_)_2_), 3.34–3.38 (m, 3H, CH(CH_3_)_2_ and 2 *x* H-2), 3.73 (t, *J* = 7.3 Hz, 2H, 2 *x* H-3), 7.50 (d, *J* = 8.4 Hz, 2H, H-3'/5'), 7.76 (d, 2H, *J* = 8.4 Hz, H-2'/6'), 9.55 (brs, 2H, NH_2_^+^). ^13^C-NMR (CDCl_3_) δ 19.4 (CH(CH_3_)_2_), 35.3, 40.2, 51.3, 129.4, 129.8, 132.3, 134.7, 195.8 (CO); MS (EI) *m*/*z*: 254 (*M* - CH_3_)^+^, 256 (*M* - CH_3_ + 2)^+^, 270.2 (*M* + H)^+^, 272.2 (*M* + H + 2)^+^. IR (KBr, cm^-1^): 2462 (NH_2_^+^), 1684 (CO). Calcd. for C_12_H_17_BrClNO (306.63): C 47.00, H 5.59, N 4.57. Found: C 46.74, H 5.52, N 4.59 (Mete *et al.*, 2011).

### Refinement

The H atoms of the NH_2_ group, HN1 and HN2, were located in a difference map and refined with a distance restraint of N—H = 0.86 (1) Å. Their displacement parameters were calculated as *U*_iso_ = 1.2*U*_eq_(N1). The other H atoms were positioned geometrically with C—H = 0.93 (aromatic), 0.96 (methyl), 0.97 (methylene) and 0.98 Å (methine), and constrained to ride on their parent atoms with *U*_iso_(H) = 1.2*U*_eq_(C) or *U*_iso_(H) = 1.5*U*_eq_(methyl C).

### Crystal data

**Table:**

C_12_H_17_BrNO^+^·Cl^−^	*D*_x_ = 1.461 Mg m^−^^3^
*M**_r_* = 306.62	Melting point: 447 K
Tetragonal, *P*4_2_/*n*	Mo *K*α radiation, λ = 0.71073 Å
Hall symbol: -P 4bc	Cell parameters from 3888 reflections
*a* = 19.7122 (4) Å	θ = 2.9–26.4°
*c* = 7.1738 (2) Å	µ = 3.12 mm^−^^1^
*V* = 2787.53 (11) Å^3^	*T* = 294 K
*Z* = 8	Block, white
*F*(000) = 1248	0.15 × 0.13 × 0.11 mm

### Data collection

**Table:**

Rigaku R-AXIS RAPID-S diffractometer	2836 independent reflections
Radiation source: Sealed Tube	1617 reflections with *I* > 2σ(*I*)
Graphite Monochromator	*R*_int_ = 0.151
Detector resolution: 10.0000 pixels mm^-1^	θ_max_ = 26.4°, θ_min_ = 2.9°
dtprofit.ref scans	*h* = −24→24
Absorption correction: multi-scan (Blessing, 1995)	*k* = −24→24
*T*_min_ = 0.632, *T*_max_ = 0.709	*l* = −8→8
50060 measured reflections	

### Refinement

**Table:**

Refinement on *F*^2^	Primary atom site location: structure-invariant direct methods
Least-squares matrix: full	Secondary atom site location: difference Fourier map
*R*[*F*^2^ > 2σ(*F*^2^)] = 0.076	Hydrogen site location: inferred from neighbouring sites
*wR*(*F*^2^) = 0.181	H atoms treated by a mixture of independent and constrained refinement
*S* = 1.07	*w* = 1/[σ^2^(*F*_o_^2^) + (0.0581*P*)^2^ + 4.3122*P*] where *P* = (*F*_o_^2^ + 2*F*_c_^2^)/3
2836 reflections	(Δ/σ)_max_ < 0.001
153 parameters	Δρ_max_ = 0.67 e Å^−^^3^
2 restraints	Δρ_min_ = −0.81 e Å^−^^3^
0 constraints	

### Fractional atomic coordinates and isotropic or equivalent isotropic displacement parameters (Å^2^)

**Table:**

	*x*	*y*	*z*	*U*_iso_*/*U*_eq_	
Br1	0.66757 (4)	0.19433 (5)	−0.04514 (15)	0.1156 (4)	
O1	0.5235 (2)	0.0463 (2)	0.7192 (7)	0.0850 (17)	
N1	0.6128 (2)	−0.1377 (2)	0.8767 (7)	0.0540 (17)	
C1	0.6340 (3)	0.0359 (3)	0.3195 (9)	0.065 (2)	
C2	0.6561 (3)	0.0740 (3)	0.1698 (10)	0.073 (3)	
C3	0.6384 (3)	0.1411 (3)	0.1604 (10)	0.069 (2)	
C4	0.5995 (3)	0.1707 (3)	0.2962 (10)	0.074 (3)	
C5	0.5762 (3)	0.1323 (3)	0.4438 (9)	0.067 (2)	
C6	0.5940 (3)	0.0644 (3)	0.4585 (8)	0.057 (2)	
C7	0.5686 (3)	0.0245 (3)	0.6205 (9)	0.061 (2)	
C8	0.5994 (3)	−0.0435 (3)	0.6592 (8)	0.063 (2)	
C9	0.5742 (3)	−0.0744 (3)	0.8392 (9)	0.061 (2)	
C10	0.5984 (3)	−0.1725 (3)	1.0610 (8)	0.0583 (19)	
C11	0.6462 (3)	−0.2324 (3)	1.0768 (9)	0.070 (2)	
C12	0.5251 (3)	−0.1928 (3)	1.0763 (8)	0.069 (2)	
Cl1	0.75617 (8)	−0.07602 (8)	0.9322 (2)	0.0682 (6)	
H1	0.64610	−0.00960	0.32750	0.0780*	
HN1	0.604 (4)	−0.168 (3)	0.794 (9)	0.1390*	
HN2	0.6548 (13)	−0.126 (4)	0.877 (12)	0.1390*	
H2	0.68250	0.05440	0.07690	0.0870*	
H4	0.58890	0.21660	0.28900	0.0890*	
H5	0.54840	0.15200	0.53360	0.0800*	
H8A	0.64830	−0.03880	0.66520	0.0760*	
H8B	0.58880	−0.07390	0.55690	0.0760*	
H9A	0.58050	−0.04270	0.94100	0.0730*	
H9B	0.52610	−0.08460	0.82920	0.0730*	
H10	0.60870	−0.14060	1.16200	0.0700*	
H11A	0.63550	−0.26500	0.98190	0.1050*	
H11B	0.64120	−0.25290	1.19730	0.1050*	
H11C	0.69210	−0.21710	1.06120	0.1050*	
H12A	0.51320	−0.22120	0.97260	0.1030*	
H12B	0.49710	−0.15300	1.07580	0.1030*	
H12C	0.51820	−0.21730	1.19040	0.1030*	

### Atomic displacement parameters (Å^2^)

**Table:**

	*U*^11^	*U*^22^	*U*^33^	*U*^12^	*U*^13^	*U*^23^
Br1	0.0916 (6)	0.1139 (7)	0.1414 (9)	0.0018 (4)	0.0247 (5)	0.0597 (6)
O1	0.091 (3)	0.074 (3)	0.090 (3)	0.020 (2)	0.021 (3)	0.008 (3)
N1	0.055 (3)	0.049 (3)	0.058 (3)	0.002 (2)	−0.001 (2)	0.007 (2)
C1	0.061 (4)	0.055 (4)	0.080 (4)	0.009 (3)	−0.003 (3)	0.007 (3)
C2	0.067 (4)	0.067 (4)	0.084 (5)	0.003 (3)	0.006 (3)	0.011 (4)
C3	0.049 (3)	0.070 (4)	0.087 (5)	−0.006 (3)	−0.004 (3)	0.023 (4)
C4	0.075 (4)	0.056 (4)	0.090 (5)	0.002 (3)	−0.008 (4)	0.013 (4)
C5	0.072 (4)	0.051 (3)	0.077 (4)	0.005 (3)	−0.003 (3)	0.000 (3)
C6	0.062 (4)	0.050 (3)	0.060 (4)	−0.002 (3)	−0.010 (3)	0.004 (3)
C7	0.056 (4)	0.060 (4)	0.068 (4)	0.000 (3)	−0.004 (3)	−0.001 (3)
C8	0.064 (4)	0.061 (4)	0.065 (4)	0.006 (3)	0.001 (3)	0.009 (3)
C9	0.060 (4)	0.053 (3)	0.070 (4)	0.003 (3)	−0.003 (3)	0.005 (3)
C10	0.068 (4)	0.056 (3)	0.051 (3)	−0.003 (3)	−0.004 (3)	0.005 (3)
C11	0.070 (4)	0.072 (4)	0.067 (4)	0.009 (3)	−0.008 (3)	0.013 (3)
C12	0.067 (4)	0.075 (4)	0.064 (4)	−0.004 (3)	0.008 (3)	0.006 (3)
Cl1	0.0608 (9)	0.0857 (11)	0.0580 (9)	−0.0135 (7)	0.0045 (7)	−0.0045 (8)

### Geometric parameters (Å, °)

**Table:**

Br1—C3	1.899 (7)	C10—C11	1.515 (8)
O1—C7	1.215 (7)	C1—H1	0.9300
N1—C9	1.486 (7)	C2—H2	0.9300
N1—C10	1.516 (8)	C4—H4	0.9300
N1—HN2	0.86 (4)	C5—H5	0.9300
N1—HN1	0.86 (6)	C8—H8A	0.9700
C1—C2	1.381 (9)	C8—H8B	0.9700
C1—C6	1.390 (9)	C9—H9A	0.9700
C2—C3	1.370 (8)	C9—H9B	0.9700
C3—C4	1.370 (9)	C10—H10	0.9800
C4—C5	1.380 (9)	C11—H11A	0.9600
C5—C6	1.388 (8)	C11—H11B	0.9600
C6—C7	1.490 (9)	C11—H11C	0.9600
C7—C8	1.498 (8)	C12—H12A	0.9600
C8—C9	1.512 (9)	C12—H12B	0.9600
C10—C12	1.503 (8)	C12—H12C	0.9600
			
C9—N1—C10	116.2 (4)	C5—C4—H4	120.00
HN2—N1—HN1	113 (8)	C4—C5—H5	120.00
C9—N1—HN1	111 (5)	C6—C5—H5	120.00
C10—N1—HN2	107 (6)	C7—C8—H8A	109.00
C9—N1—HN2	106 (5)	C7—C8—H8B	109.00
C10—N1—HN1	105 (4)	C9—C8—H8A	109.00
C2—C1—C6	121.1 (6)	C9—C8—H8B	109.00
C1—C2—C3	118.9 (6)	H8A—C8—H8B	108.00
Br1—C3—C2	119.7 (5)	N1—C9—H9A	110.00
Br1—C3—C4	119.1 (5)	N1—C9—H9B	110.00
C2—C3—C4	121.3 (6)	C8—C9—H9A	110.00
C3—C4—C5	119.9 (6)	C8—C9—H9B	110.00
C4—C5—C6	120.2 (6)	H9A—C9—H9B	108.00
C5—C6—C7	118.9 (5)	N1—C10—H10	108.00
C1—C6—C7	122.5 (5)	C11—C10—H10	108.00
C1—C6—C5	118.6 (5)	C12—C10—H10	108.00
O1—C7—C6	120.9 (5)	C10—C11—H11A	109.00
C6—C7—C8	118.7 (5)	C10—C11—H11B	109.00
O1—C7—C8	120.3 (6)	C10—C11—H11C	109.00
C7—C8—C9	112.7 (5)	H11A—C11—H11B	110.00
N1—C9—C8	109.0 (5)	H11A—C11—H11C	110.00
N1—C10—C12	111.4 (5)	H11B—C11—H11C	110.00
C11—C10—C12	112.6 (5)	C10—C12—H12A	109.00
N1—C10—C11	107.5 (5)	C10—C12—H12B	110.00
C2—C1—H1	119.00	C10—C12—H12C	109.00
C6—C1—H1	119.00	H12A—C12—H12B	109.00
C1—C2—H2	121.00	H12A—C12—H12C	109.00
C3—C2—H2	121.00	H12B—C12—H12C	109.00
C3—C4—H4	120.00		
			
C9—N1—C10—C11	−176.0 (5)	C3—C4—C5—C6	2.3 (9)
C9—N1—C10—C12	60.1 (6)	C4—C5—C6—C1	−1.7 (9)
C10—N1—C9—C8	174.9 (4)	C4—C5—C6—C7	179.4 (6)
C6—C1—C2—C3	0.5 (9)	C5—C6—C7—C8	−166.9 (5)
C2—C1—C6—C5	0.3 (9)	C1—C6—C7—O1	−165.4 (6)
C2—C1—C6—C7	179.2 (6)	C1—C6—C7—C8	14.2 (9)
C1—C2—C3—C4	0.1 (10)	C5—C6—C7—O1	13.5 (9)
C1—C2—C3—Br1	−179.5 (5)	O1—C7—C8—C9	−6.8 (8)
C2—C3—C4—C5	−1.4 (10)	C6—C7—C8—C9	173.6 (5)
Br1—C3—C4—C5	178.1 (5)	C7—C8—C9—N1	−173.8 (5)

### Hydrogen-bond geometry (Å, °)

**Table:**

Cg1 is the centroid of the benzene ring.

**Table:**

*D*—H···*A*	*D*—H	H···*A*	*D*···*A*	*D*—H···*A*
N1—HN2···Cl1	0.86 (4)	2.26 (4)	3.102 (4)	166 (5)
N1—HN1···Cl1^i^	0.86 (6)	2.27 (6)	3.133 (5)	177 (9)
C12—H12B···O1^ii^	0.96	2.60	3.378 (7)	139
C9—H9B···Cg1^iii^	0.97	3.00	3.943 (6)	164

Symmetry codes: (i) −*y*+1/2, *x*−1, −*z*+3/2; (ii) −*x*+1, −*y*, −*z*+2; (iii) −*x*+1, −*y*, −*z*+1.
